# A Quality by Design Framework for Capsule-Based Dry Powder Inhalers

**DOI:** 10.3390/pharmaceutics13081213

**Published:** 2021-08-06

**Authors:** Li Ding, Ashlee D. Brunaugh, Sven Stegemann, Scott V. Jermain, Matthew J. Herpin, Justin Kalafat, Hugh D. C. Smyth

**Affiliations:** 1 Division of Molecular Pharmaceutics and Drug Delivery, College of Pharmacy, The University of Texas at Austin, Austin, TX 78712, USA; dingli@utexas.edu (L.D.); ashbrunaugh@gmail.com (A.D.B.); sjermain15@utexas.edu (S.V.J.); matt.herpin@utexas.edu (M.J.H.); 2 Institute for Process and Particle Engineering, Graz University of Technology, 8010 Graz, Austria; sven.stegemann@tugraz.at; 3 ACG North America, LLC, 262 Old New Brunswick Road, Suite A, Piscataway, NJ 08854, USA; justin.kalafat@acg-world.com

**Keywords:** quality by design, inhalation capsule, dry powder inhalers, capsule activation, capsule manufacturing, capsule filling, capsule storage

## Abstract

Capsule-based dry powder inhalers (cDPIs) are widely utilized in the delivery of pharmaceutical powders to the lungs. In these systems, the fundamental nature of the interactions between the drug/formulation powder, the capsules, the inhaler device, and the patient must be fully elucidated in order to develop robust manufacturing procedures and provide reproducible lung deposition of the drug payload. Though many commercially available DPIs utilize a capsule-based dose metering system, an in-depth analysis of the critical factors associated with the use of the capsule component has not yet been performed. This review is intended to provide information on critical factors to be considered for the application of a quality by design (QbD) approach for cDPI development. The quality target product profile (QTPP) defines the critical quality attributes (CQAs) which need to be understood to define the critical material attributes (CMA) and critical process parameters (CPP) for cDPI development as well as manufacturing and control.

## 1. Introduction

Dry powder inhalers (DPIs) are widely utilized for the treatment of multiple lung diseases including asthma [1], chronic obstructive pulmonary disorder (COPD) [2], cystic fibrosis (CF) [3], and CF-related *Pseudomonas aeruginosa* infections [4], virus-related lung infections [5] and systemic diseases like diabetes [6]. While various dose metering systems have been developed for DPIs, including blisters or reservoir-based devices [7], capsule-based DPIs (cDPIs) remain an important system for the therapeutic delivery of inhaled powders, with half of all DPIs on the market using this dose metering mechanism [8] (Table 1). cDPIs have been shown to provide accurate and consistent drug delivery [9] with multiple patient feedback mechanisms (e.g., visual, auditory) to assure that the dose was delivered [10].

**Figure 1:**
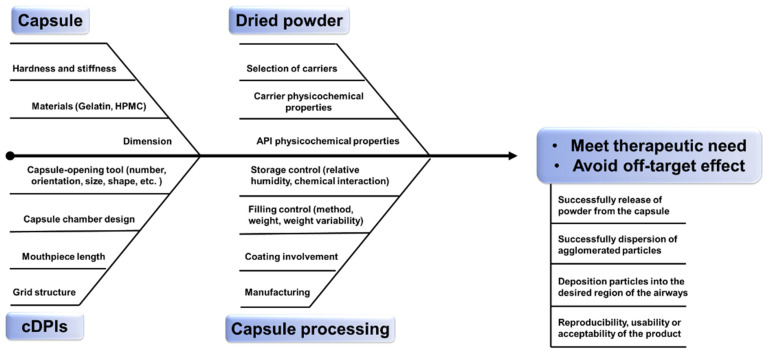
Quality by Design (QbD) framework of cDPIs.

The successful delivery of therapeutics from cDPI delivery systems involves a complex interplay of factors associated with the powder formulation, the formulation-capsule, and the device-capsule interactions. Consistency and predictability of the delivered dose to the patient are of great importance in product development and manufacturing of new chemical entities (NCE) or generic products. Demonstrating bioavailability and/or bioequivalence of DPI products remains an important product attribute for drug approval by the regulatory authorities [20]. Apart from pharmacologic reasons, failures in the development of NCE or generic inhaled therapies can stem from either a lack of understanding about aspects of the drug/formulation powder [21], device [22,23,24], and/or the mechanisms by which they interact. Several reviews have been published on DPI formulation design [25] or engineering strategy [26] as well as how the design [27] and characteristics [28] of DPI devices affect powder aerosolization performance revealing the multifactorial challenge of DPI products. This review seeks to expand these previously published analyses by providing insights into the critical material attributes (CMA) and critical process parameters (CPP) and hence critical quality criteria (CQA) to be considered for cDPI development and manufacture (Figure 1). Especially it relates to the inclusion of the capsule and device component as well as its metering system in order to provide further guidance on a QbD approach for the development of NCE or generic cDPI products.

## 2. Overview of Quality by Design Approach for cDPIs

As defined by the International Council for Harmonization’s Q8R2 guideline, QbD is a “systematic approach to development that begins with predefined objectives and emphasizes product and process understanding and process control, based on sound science and quality risk management” (ICH Q8R2) [29]. A QbD approach is intended to generate sufficient product and process understanding that the robustness of the manufacturing process and the reproducibility of the clinical performance of the final drug product is assured. The basis of a typical QbD approach involves a clearly defined quality target product profile (QTPP), followed by a risk assessment in order to identify potential CQAs, CMAs, and CPPs of the process or product. The QTPP provides a comprehensive summary of all the required targets that will ensure the quality, safety, and efficacy of a specific product for the patient. The CQAs are the properties or characteristics of the product that should be within an appropriate limit, range, or distribution to ensure the desired product quality and performance (ICH Q8) [30]. The CMAs of the input materials and their properties can be identified, optimized, and controlled to ensure the desired quality of output materials [30]. A process parameter whose variability has an impact on a CQA and therefore should be monitored or controlled is termed as CPPs (ICH Q8) [30]. In addition, a number of design of experiments (DoEs) are performed to further delineate the design space and associated control strategies [31].

**Figure 2:**
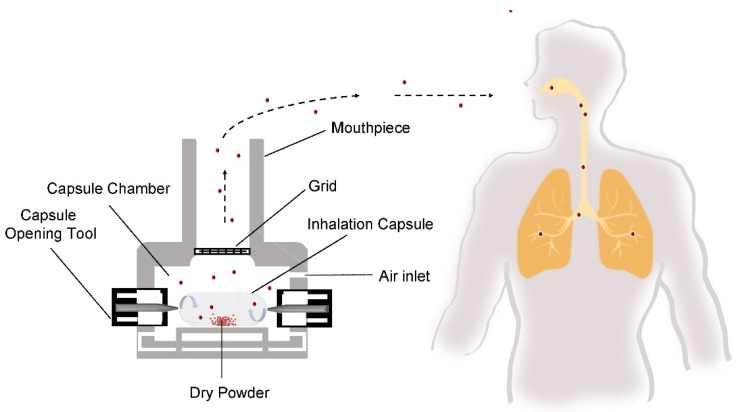
Schematic of an example of successful delivery of the drug to the lungs.

The delivery of drugs to the lungs via cDPI carries a unique set of quality and performance criteria that are unique to the pulmonary route of delivery. Depending upon the therapeutic indication, cDPIs may be utilized for the delivery of a variety of formulation systems including a binary mixture of large carrier particles and micronized drug particles, a carrier-free, high-dose (>5–10 mg) drug formulations [32], or engineered particle using process technologies like spray drying [33]. Successful delivery of the drug to the lungs via cDPI involves the completion of several steps (Figure 2), including (1) capsule opening or piercing by the device, (2) release of powder from the capsule, (3) entrainment in the airflow, (4) dispersion of deaggregation of particles into primary particles or separation of carrier particles and drug particles, and (5) deposition of the drug particles into the desired region of the airways. The reproducibility of each of these steps and subsequently the reproducibly of the delivered dose and therapeutic effect is dependent upon patient-associated factors, such as inspiratory force or correct actuation of the device, as well as product factors like the initial raw material properties of the formulation components (e.g., particle shape, size, surface properties, crystallinity, moisture content of the excipients and drug) and capsule, the processing approach (e.g., milling, blending, spray drying, capsule filling), and the packaging and storage of the cDPI product.

Essentially, the QTPP of cDPIs requires that the therapeutic need of the patient be met while avoiding off-target drug effects or toxicities. It is well known that lung deposition of therapeutic particles is challenging and depends upon many factors including drug formulation factors, device, patient parameters, and also the disease state. The requirement for systemic or targeted/topical therapeutic effect must also be considered with regard for the desired location of drug deposition. In case cDPIs are being used to achieve systemic therapeutic effects, it is likely to require deposition in the large-surface area, capillarity-rich region of the alveoli [34]. In fixed-dose combination therapy cases, where a synergistic effect is required the deposition efficiency and location of each drug in the formulation are even more important [35].

The approach to the pharmaceutical development described by the ICH Q8 (R2) guideline states that: “In all cases, the product should be designed to meet patients’ needs and the intended product performance.” Consequently, in addition to pharmacologic-based adverse drug reactions or toxicities, any other factor that reduces the usability or acceptability of the product by the patient should be considered [36]. For example, detachment of capsule pieces and subsequent inhalation by the patient may result in fragment deposition in the throat [37]. Variability in aerosol performance may result in unintended off-target effects of potent drugs, as noted in the case of inhaled insulin [38] or gene therapy agents [39]. Likewise, an unexpected increase in oropharyngeal deposition of drugs that are intended for lung-targeted therapeutic effects may result in the occurrence of systemic side effects [40].

## 3. Critical Material Attributes (CMAs) in cDPIs

As with other inhaled drug delivery systems, the critical CQAs of cDPIs to achieve the aforementioned QTPP are related to the delivery of a consistent fine particle dose of the drug in a manner that is robust to deviations in patient-related factors. These CQAs are in turn dependent upon CMAs associated with the cDPI, which should be controlled or limited [41]. Specific to cDPIs, CMAs relate to API and excipients including the material properties and design of the capsule, the capsule piercing/powder release mechanism present in the device, and the interaction of the powder formulation with the capsule.

### 3.1. CMAs Related Capsule Properties

#### 3.1.1. Capsule Material

Typically, cDPIs use hard-shell capsules to deliver dry powders to the lung. The hard-shell capsule is comprised of two open-ended cylinders called the cap and the body. The cap fits over the body to form the complete hard shell capsule [42]. The hard-shell capsules utilized in cDPIs are typically prepared from either gelatin or hydroxypropyl methylcellulose (HPMC). Each polymer has unique properties which must be considered in product development (Table 2).

Hard gelatin capsules are made of gelatin and eventually dyes, but do not include any plasticizer. If stored at temperatures between 15–25 °C and 30–65% relative humidity (RH), hard gelatin capsule shells will have a moisture content (determined by loss on drying (LOD)) of 13–16% water due to the hysteresis properties of the gelatin polymer [43]. In these conditions, the capsule can maintain rigidity and elongation [44]; however, if the water content falls below 13% due to environmental factors (e.g., <30% RH or hygroscopic formulation), the shell will gradually lose its shell flexibility and become brittle [42]. On the other hand, if the water content is above 16%, the shells will become soft and sticky [42]. Consequently, alterations in the capsule moisture content may have effects on various aspects including effects on the API-containing powder which may alter the overall product stability and performance. In this regard, it is important to consider that the capsule moisture content is dynamic and directly related to the environmental storage condition with equilibration occurring within hours.

In contrast to hard gelatin capsule shells, HPMC capsule shells typically have a water content of less than 9% [45] under the standard storage conditions of 15–25 °C and 30–65% RH. The differences in water content between gelatin and HPMC capsule materials can affect aerosol performance and lung deposition under certain circumstances. For example, the encapsulation of hygroscopic drugs in hard gelatin capsules may result in increased water loss from the capsule and subsequently changes in the powder properties (e.g., increased agglomeration) as well as brittleness of the capsule [46]. Both may result in reductions in the fine particles fraction (FPF) and deep lung deposition of the drug [23]. In general, HPMC capsule shells seem to be less sensitive to moisture transfer and low water content. Pre-equilibration of both the powder and capsule at the same relative humidity prior to filling can potentially reduce these effects, as discussed in a later section. The selection of the manufacturing process of HPMC capsules (cold-gelling or thermal-gelling) can also affect aerosol performance. Capsules manufactured by cold-gelling use a gelling system (e.g., carrageenan/potassium chloride) leading to a slightly rougher internal capsule surface, while thermal-gelled capsules are composed without a gelling system providing a smooth, glossy surface similar to that of hard gelatin capsules [47]. The importance of the surface roughness of the capsule wall has not been fully studied in inhalation systems. The physical impaction of particles with the rough capsule wall has been shown to increase the powder-wall friction by using calcium carbonate and maize starch as experimental drugs [48]. Furthermore, the increased friction can induce uncontrollable powder adhesion to the interior capsule wall [48], which results in the reduction of the emitted dose (ED) [49].

The utilization of gelatin versus HPMC must be carefully considered with regard to some key targets in the QTPP of the cDPI due to the differences in capsule composition and material characteristics. The first gelatin-based capsules used for dry powder inhalers just appeared 50 years ago, which was also the first commercially successful cDPI, whose application was to deliver cromolyn sodium (sodium cromoglycate) by using two pins to puncture the capsules [50]. Until today the evolution of technology and performance of the cDPI systems continues, whereby gelatin capsules are still leading the market due to the ease and availability of capsule filling [51]. Compared to hard gelatin capsules, the recently introduced HPMC capsules have been demonstrated to have several advantages such as requiring less force for shell puncture [52,53,54] which results in a more regular aperture and less shedding of pieces [55]. This has been correlated to an improved delivered dose, fine particles dose, and less powder retention in the HPMC capsules relative to gelatin capsules [56]. The superior piercing characteristics of HPMC capsules may be related to the reduced brittleness of the shell, particularly when exposed to ambient conditions of less than 30% RH [57]. Moreover, gelatin capsules were found more susceptible to loss of the shell “flap” created upon piercing by the device [23,58]. These “flaps” may be important for the aerosol performance of dry powders since they can potentially change the airflow profile and release from the capsule, as well as that powder could stick to them and not fully get to the patient during inspiration [59]. However, their precise effects on powder de-agglomeration are still poorly understood and require further investigation. To reduce the brittleness of gelatin capsules, 4–6% of a low molecular weight PEG was added to the gelatin. While the addition slightly reduced the brittleness and improved the piercing, HPMC capsules performed superior in terms of stability and piercing under low moisture conditions [60].

A study from Telko et al. used a statistical experimental design to look specifically at capsule material type effects on powder charging, which is thought to correlate with aerosol performance by affecting powder detachment from surfaces of capsule walls [61]. The authors determined the choice of the capsule (gelatin vs. HPMC) has a large effect on the polarity of the charge but only a minor effect on the magnitude of the charge from the powders. They found the use of HPMC-based capsules led to a higher triboelectric charging of the powder than gelatin-based capsules. However, it has been observed in other studies that a higher potential for triboelectrification was observed in gelatin capsules when compared to HPMC capsules [62], and therefore this effect should be further evaluated and might depend much more on the moisture content of the shells and the formulation itself.

#### 3.1.2. Capsule Dimension

Capsule dimensions are based on their respective fill capacities, which in turn are based on the tapped density of the dry powder [63]. Capsule dimension is represented by a numerical size assignment, with larger numerical sizes representing a smaller internal capsule volume that ranges numerically from sizes 000 (the largest size) to 5 (the smallest size) [45]. While capsule size 3 is the standard cDPI capsules, larger and smaller capsule sizes are being evaluated for high-dose drugs or potent APIs. It should be noted that even though the capsule sizes are standardized, capsule dimensions can vary between suppliers. Especially, the close length of the capsule might be important for the piercing performance as well as the movements within the capsule chamber. In this respect, it needs to be assured that the capsules are closed properly and provide sufficient closing strength preventing reopening when pierced at the hemispherical ends. When considering general capsule motion from within a fixed capsule chamber, the smaller capsules may be thought to have more free space and thus can move freely in a turbulent fluid flow. Additionally, there is a long distance for the capsule to travel to impact the chamber walls, which may reduce the number of possible collisions in a given time compared to larger capsules that fit more squarely inside the device chamber. This was illustrated in a study performed by Coates et al., in which the overall levels of turbulence within the device were found to diminish with the increase in capsule size [64]. The higher frequency of collisions noted with the larger capsule sizes resulted in increased device powder retention after inhalation. However, while smaller capsules may demonstrate the release of dry powder from the device, this must be balanced with the reduced carrying capacity of the capsule, which may require an increased number of doses loading and inhalation maneuvers by the patient for a therapeutic effect to be achieved.

#### 3.1.3. Capsule Hardness and Stiffness

The stiffness and hardness of the capsule can influence the cDPI performance in multiple ways. As described previously, stiffness and hardness alteration of the capsule due to a change in moisture content can affect the capsule piercing characteristics. Harder and stiffer capsule shells require increased puncture force, which may have negative influences on aerosol performance [65]. Additionally, the hardness of the capsule is thought to be a factor that controls the collision velocity between capsules and inhaler walls [53]. When the capsule collides with the inhaler wall with high collision speeds and frequency a considerable impaction force is generated. A capsule with softer shell walls buffers a portion of the impact force, causing the velocity of the capsule motion to decrease. In turn, this leads to a lower collision frequency and alters capsule motion, which further fails to de-aggregate the powder [66] and achieve acceptable powder release [67]. On the other hand, if the capsule is too soft, the high velocity of capsule-inhaler wall collisions can induce macroscale capsule deformation, thus altering the capsule surface from a smooth, flat morphology to a rough morphology and thereby promoting increased powder retention in the capsule [53].

#### 3.1.4. Others

The weight ranges of capsules must be narrower for inhalation than standard powder filling due to the low fill weights and potential rejections on the high-speed encapsulators [68]. And the microbial limits on inhalation capsules must be lower due to the drug product inside being delivered directly to the lungs.

Altogether, the capsule-related CMAs lead to the finding that capsule dimension should balance powder payload and aerosol performance, and the moisture contents of the inhalation capsule need to be controlled within a narrow range.

### 3.2. CMAs Related cDPIs Device Design

Powder deaggregation level has been widely proven to be correlated to the design of the cDPI device [69,70]. Most marketed cDPIs devices contain four common design features to facilitate powder deaggregation, including grid structure, mouthpiece, capsule chamber, and capsule-opening tool. The following context will discuss these components one by one of their impact on the QTPP performance parameter of cDPIs.

#### 3.2.1. Grid Structure

The grids are important for efficient powder deaggregation based upon impaction between the grid and powder during inhalation. The effects of grid design specifically on aerosol performance of cDPIs were examined by Coates et al. [71]. Using computer fluid dynamics (CFD), it could be shown that the grid significantly impacts the flow turbulence levels and particle impaction velocities which triggers the deagglomeration of the DPI powder.

#### 3.2.2. Mouthpiece Length

The mouthpiece length has been stated to determine the airflow development inside the mouthpiece and exiting the cDPIs [71]. Ideally, a more developed flow profile at the mouthpiece exit can potentially increase the oropharyngeal deposition of cDPIs after actuation. However, from the study given by Coates et al., there is no significant change to FPF if reducing the length of the mouthpiece from the original length to three-quarters and one-half. And minimal difference (6.7 and 6.2%, respectively vs. 4.9%) can be found in the throat impaction among comparison. This conclusion has also been confirmed by Tuteric et al. [72]. They modified the Aerolizer device to different lengths of the mouthpiece and found that higher velocities of flow field were present with mouthpiece length increased. Moreover, they also stated that the modified cDPI which has the longest mouthpiece length exhibited the highest level of deposition.

#### 3.2.3. Capsule Chamber Design

In practice, when patients are using a passive cDPI, the inhalation maneuver begins after the capsule has been pierced. In some cases, when the airflow enters the capsule chamber, the capsule moves and may repeatedly collide with the inhaler walls and internal structures [73]. While the amount of research in such capsule motion is limited, a recent study was conducted by Benque et al., which examined capsule rotation in the Aerolizer device using high-speed photography, computational fluid dynamics, and discrete element method (DEM) simulations [74]. The authors found that the amount of capsule rotation produced from different flow rates (between 30 and 100 L/min) was crucial to capsule powder dispersion and that increased capsule collisions vastly improved the discharge of polydisperse powders. Therefore, different capsule chamber designs, which are associated with different capsule motion induction, can also affect the performance of the cDPIs.

Several design iterations of capsule chambers have been featured in a commercially available device, and the differences in these designs have been linked to differences in aerosol performance of cDPIs. For example, the Dinkihaler^®^ (Aventis, GA, USA), the capsule ends are fitted into an impeller-shaped capsule chamber from which powder is released to rotate during inspiration [69]. It spins under the inspirational airflow, and the rotational speed depends on the inspiratory force and breathing cycle which determines the rate of aerosolization and dispersion. In contrast, the Rotahaler^®^ is activated by separating the powder-containing capsule body from the capsule cap into the barrel chamber. During inhalation, powder emission mechanisms from the Rotahaler, regulated by its impaction on the grid and the Rotahaler wall as well as the rotational movement in the entrained air, contributing to the de-agglomeration of the drug powder [75]. Chew and colleagues compared these two devices with different capsule chamber designs and found that Dinkihaler^®^ DPI gave much higher FPF [69].

Different capsule chamber designs can also determine the capsule position once loaded into the capsule cavity, which may further affect the performance of the cDPIs. The study given by Behara et al., compared two different experimental capsule device chamber designs, in which the long axis of the capsule has oriented either perpendicular or aligned with the airflow passage. The perpendicular design was found to increase the vibrational frequency of the capsule compared with capsules aligned with the flow, which increased the deaggregation of the dry powder and resulted in a smaller MMAD, but more powder retention in the capsule [76]. Recently, the vertical aerosolization chamber design along with the aforementioned 3D array design has been reported to be applied in a positive pressure cDPI for children, which showed advantageous lung delivery efficiency [77]. Moreover, whether the angle between the capsule chamber and the flow passage affects aerosol performance has also been studied. In another study given by Behara et al., they compared inhalers that implement either a 45° or 90° angle designed cDPIs [78]. However, no significant differences have been discovered regarding aerosol performance.

As the example of commercialized cDPI devices, the long axis of Cyclohaler^®^ (with a similar design to the Aerolizer^®^ in the USA) is aligned with the tangential direction, while HandiHaler^®^ is different which enable capsule to be inserted in the vertical position, and rest over the chamber inlet. Shur et al., examined pressure and velocity distributions in the capsule chamber of the HandiHaler^®^ and Cyclohaler^®^. This supplied a near comprehensive view of the capsule motion inside these two inhalers respectively [73]. For the HandiHaler^®^, the sequential high-speed video images revealed the axial vibration movement sustained throughout the operation of the device, which impels the powder to exit the capsule. Conversely, due to the distinguished structure of Cyclohaler^®^, swirling or cyclonic flow structure dominated the flow pattern inside the Cyclohaler^®^, which resulted in the capsule moving across a rotational axis thus forcing the capsule rotation [73]. Consequently, while they have a comparable median mass aerodynamic diameter (MMAD), ED of the tested dry power obtained by Cyclohaler^®^ is 14% and 15% less compared to HandiHaler^®^ at 20 and 39 L/min air flow rate, respectively [73].

#### 3.2.4. Capsule-Opening Mechanisms

**Figure 3:**
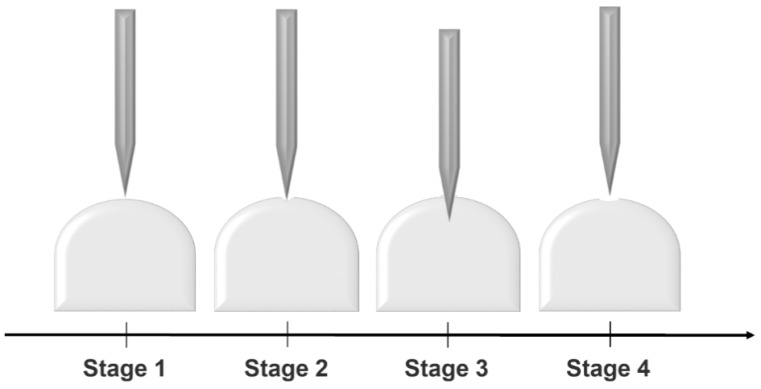
Puncturing process of the capsule. Stage 1: the initial interaction of the pin and the capsule. Stage 2: the pin expose forces to the intact capsule shell. Stage 3: the pin punctures the capsule shell and keeps entering. Stage 4: the pin is removed from the pierced capsule.

Several capsule-opening tool designs have been developed to facilitate capsule opening and powder release, including piercing the capsule shell with sharpened pins, shear opening, or cutting it with blades [51,55]. In this review, only the pin-based capsule open mechanism will be discussed due to the availability of published data on these designs. For pin-based capsule activation devices, four identified stages make up the puncturing event (Figure 3). Stage 1 is the initial interaction of the pin and the capsule, with the rapidly increasing piercing force and deformation of the capsule. Stage 2 involves the puncturing of the capsule shell resulting in a sharp reduction of the pin forces. Stage 3, in which the pin keeps penetrating through the shell, is characterized by the progressive reduction in the puncturing force. At this stage, the resistance to the piercing mostly comes from the frictional forces between the surface of the pin and the outside edge of the punctured aperture in the capsule, including any flaps. Finally, Stage 4 is the removal of the pin from the capsule [53].

Capsule opening is crucial to the successful delivery of the powder from the capsule, since capsule piercing characteristics associated with aperture (aperture shape, size, orientation, number) can affect the emitting manner of dry powder inside the capsule substantially. In addition, the orifices of capsules can also change the nature of the airflow dynamics, in manners such as altering the turbulence inside as well as outside the capsule. This affects the capsule motion in the device and the release and/or retention of powder in the capsules [76,79]. Therefore, discussion on distinctive piercing characteristics caused by differences in pin (number, orientation, size, shape) has been widely studied.

##### Pin Number

Saleem et al., looked at the influence of pin number by using DPIs (with a different number of punctures (2-pins vs. 8-pins) [80]. A significant difference was found using HPMC capsules between 8-pin and 2-pin DPI devices, demonstrating that the 2-pin DPI device showed significantly lower MMAD in comparison with the 8-pin DPI device Another study conducted by Torrisi et al., looked at the number of punctures (2 sets of 4-pins vs. 2 single pins DPI) with gelatin and HPMC capsules, indicating that a greater mean force was needed for 2 sets of 4-pins penetration in Stage 1 and Stage 2 puncturing event mentioned before in both HPMC and gelatin capsules compared to single pin penetration [53].

##### Pin Orientation

**Figure 4:**
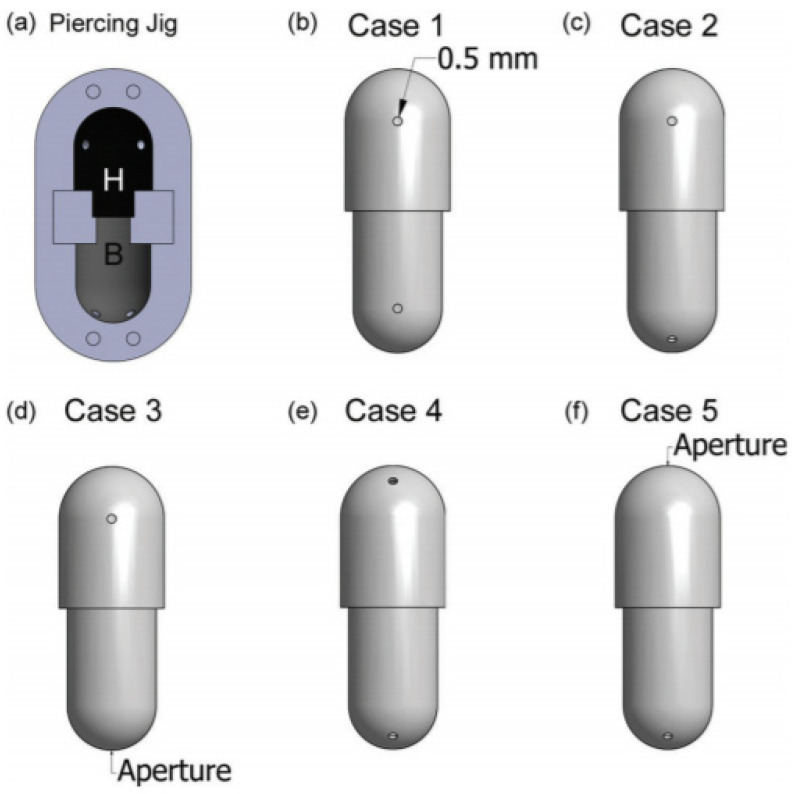
Five different cases of capsule aperture orientations generated by capsule jig piercing at different orientations. Reproduced with permission from [78], Elsevier, 2014.

Behara et al., conducted the experiments for the assessment of the effect of pin orientation by where a custom capsule jig was used to create a capsule with different aperture orientations. They compared five different cases of capsule aperture orientations (Case 1: the start of top curvature as air inlet aperture and the start of bottom curvature as air outlet aperture; Case 2: the start of top curvature as air inlet aperture and the middle of bottom curvature as air outlet aperture; Case 3: the start of top curvature as air inlet aperture and the center of the bottom dome as air outlet aperture; Case 4: the middle of top curvature as air inlet aperture and the middle of bottom curvature as air outlet aperture; Case 5: the center of the top dome as air inlet aperture and the middle of bottom curvature as air outlet aperture) [78] (Figure 4). As a result, although capsules with Case 1 aperture orientation showed the most capsule powder retention proportion and the smallest capsule inhaler retention proportion, no significant difference was found on ED or other inhalation metrics among capsules with Case 1 to Case 3 aperture orientation. This suggests that the air outlet aperture orientation has little importance to the device’s performance. Another comparison between capsules with Case 2, Case 4, and Case 5 aperture orientation showed that capsules with Case 2 aperture orientation exhibited better aerosol performance, indicating that the start of top curvature’s air inlet aperture probably is the most optimal strategy for inhalation. Interestingly, Shur et al., reported a similar result. They found that the air inlet orifice of the capsule can produce a high-velocity air jet through the CFD model of the HandiHaler^®^ [73]. The authors concluded that the location of the air inlet aperture of the capsule, which is associated with pin orientations, was of great significance for optimal fluid flow.

##### Pin Size

Pin size can decide the capsule pierced orifice size after actuation. Capsule orifice size may be linked with powder residence time within the capsule depending on the device, formulation, and inhalation effort, and may facilitate powder de-agglomeration as well as powder emptying [64]. Son et al., compared the energy available for the powder dispersions (*E*_dispersion_) and powder dispersion characteristics of two 1.5 mm orifice versus a 0.5 mm orifice created by HandiHaler^®^ needles, showing that, though the capsule with 0.5 mm pierced aperture required more E_dispersion_, it had a larger FPF less than 5 μm in diameter (FPF < 5 μm), while having a lower MMAD and ED [81]. This indicates that the optimized aperture size is likely to be in a range that allows for both sufficient de-agglomeration of powder and minimizes powder retention. With a large aperture, agglomerates will easily emit from the capsule but will result in large particles sizes at the same time. However, significant powder retention can be caused by an aperture that is too small [82]. For different cDPIs, this optimal size range can be quite diverse. For the Dinkihaler^®^, the optimal orifice size of the capsule range was found to be 1.00 and 2.38 mm, with the favorable FPF < 5 μm of 50–60% by mass with significantly less capsule and device powder retention [83]. Another study given by Behara et al., suggested that the size of the capsule aperture should be around 0.5 mm. This was done by comparing three different aperture sizes of 1.5, 0.8, and 0.5 mm punctured by HandiHaler^®^ needles in order to maximize FPF but minimize the MMAD [76]. Behara et al., also had another independent study that accurately calculated the de-agglomeration rate (*k_d_*) and found that a decrease in capsule aperture size increased the *k_d_* [84]. Therefore, optimal capsule aperture size should be a range, instead of a definite number and will vary between the different inhaler devices in order to meet both the satisfied aerosol performance and minimized powder retention.

##### Pin Shape

There are few studies about the effect of pin shape, one representative example is given by Torrisi et al. [53]. In their study, a conical pin, which was described as a decreasing diameter rod ending in a rounded pin tip, and an angular pin, which showed as a flattened structure ending in a point, have been used for comparison. As a result, differences have been investigated within both Stage 2 and Stage 3 puncturing events. At the terminal point of Stage 2, interestingly, the recorded force for angular pin puncture did not reduce to 0 N, as the conical pin did, indicating that angular pin puncture causes the formation of flap attached to the capsule walls [53]. The reason might be the structural differences between these two pins. The rounded pin tip of the conical pin was suitable for puncturing the shell wall, while the beveled pin tip tended to cut the capsule shell wall like a blade to penetrate. The recorded forces and force duration of Stage 3 were found to be greater for the angular pin in comparison to the conical pin, which is attributed to the longer tapered portion of the angular pin. These pin shape-induced puncture profiles differences resulted in aperture shape and flap attachment differences. While from the investigation of this study, angular pin tended to form irregular aperture shape and cause flap retention after piercing [53]. Having capsules with irregular apertures increases the possibility of causing the cracked or fractured capsules pieces, which further increase the aperture structure and size to affecting the performance criteria of cDPIs as mentioned above.

**Figure 5:**
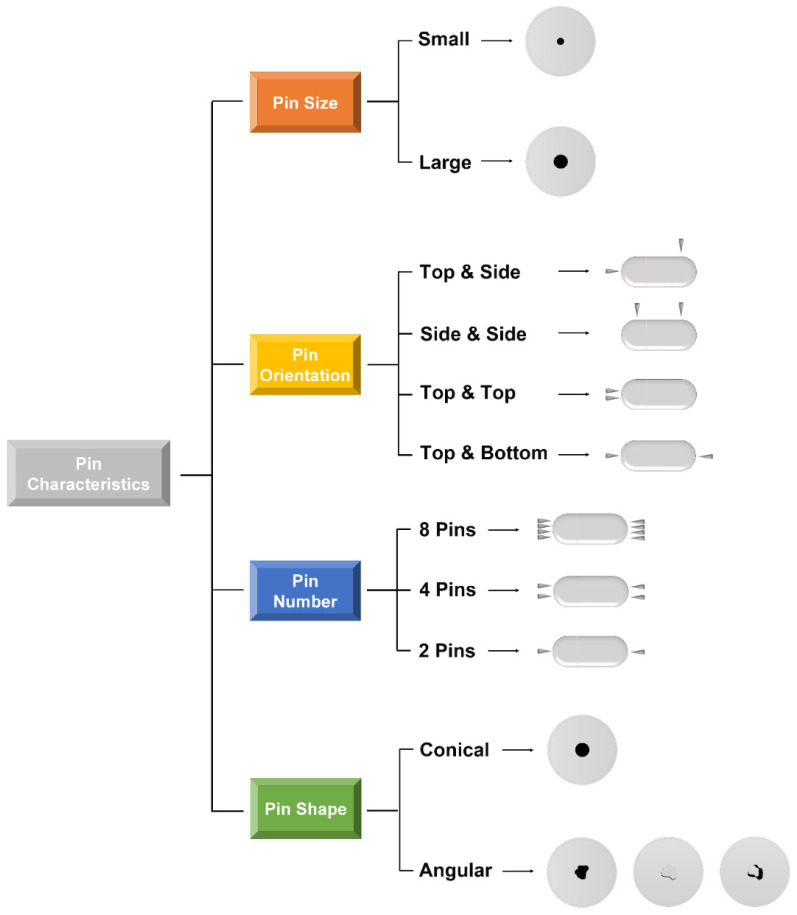
The pin characteristics category within pin activated cDPIs and their outcome on capsule appearance after piercing.

In all, the capsule-opening mechanism as described for the pin-based capsule activation devices, reveal a complex interaction between the pin design, capsule attributes, and device factors which finally require investigation and eventually optimization of pin shape, number, orientation, and size for each product (Figure 5).

### 3.3. Dry Powder Related CMAs

A key CQA of the cDPI product defined in the QTPP is the reproducible, targeted delivery to the lung under patient acceptable use patterns. Taking this into account, efficient delivery to the lungs requires that the inhaled dry powder should be able to bypass deposition in the upper respiratory tract, and not being exhaled when used by the patients e.g., with impaired lung functions [85].

The aerodynamic diameter (*d_ae_*) of the API or API loaded particles has been found to determine the in vivo deposition of a cDPI product [86]. It was shown that the percentage of the dry powder with *d_ae_* < 3 μm have a closer numerical equivalence to the in vivo lung deposition [86]. However, neat dry powders (*d_ae_* < 3 μm) are prone to agglomerate due to the interparticulate cohesive and adhesive forces and high surface energy [87]. Since the inspiratory flow rate plays a key role in powder deagglomeration, the physicochemical properties of the API particles or the API containing dry powder mixtures are most important for targeted lung delivery [85]. At fixed inspiratory flow rates (e.g., 30, 60, 90 L/min), the evaluation of the physicochemical properties of the dry powder provides a necessary CQA since it is associated with the de-agglomeration of the powder within the capsule during inhalation. To enhance processing and powder de-agglomeration within cDPI during inhalation, most dry powder mixtures use coarser carrier excipients, mainly lactose [88]. The drug particles in such powder mixtures adhere to the surface of the lactose particle during blending, which allows for better-flowing powders and more uniform dispersions. These carrier-based formulations transform cohesive agglomerates of drug particles in drug-carrier adhesive agglomerates. In this case, in addition to dry powder, the physicochemical properties of sugar carriers (mostly lactose) must be considered and investigated as a CMA.

#### 3.3.1. Carrier-Free Systems

Pertaining to the carrier-free systems, the *d_ae_* of the API powder exits from the cDPIs should be at around 1–3 µm for deep lung deposition. The *d_ae_* of the API particles is determined by its using equation if regardless of the effect of inspiratory flow rate [89]:

(1) $$d_{ae}=d_{geo}\surd \left(\frac{\rho_{p}}{\rho_{o}X}\right),$$

where $d_{geo}$ represents the geometric diameter of the API particle, X is the dynamic shape factor (e.g., deviation from API particle sphericity), $\rho_{p}$ and $\rho_{o}\;$ are the API particle and unit densities, respectively. From the equation, the geometric size [90] and density [91] of the API particles can result in significant changes in performance. For example, high porosity (low density) API particles do tend to distribute in deeper pulmonary tissue [92]. And one study from Brunaugh et al., found that decreasing the size of clofazimine by jet milling can significantly increase the aerosol performance of this excipient-free formulation [93]. More detailed information about other factors can be found in Table 3.

**Figure 6:**
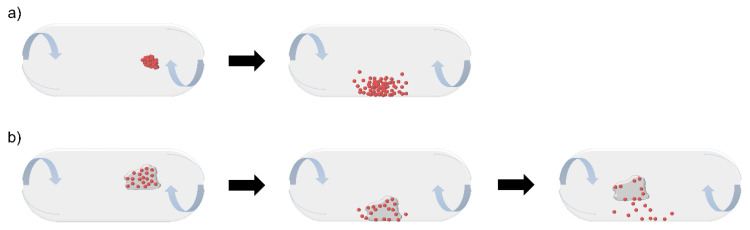
Schematic illustration of de-agglomeration mechanism within the capsule for (**a**) carrier-free agglomerates and (**b**) carrier-based formulation.

The interparticle interactions of pure API-associated powder are characterized by higher surface energy which are important factors for the physicochemical properties [94] besides a decreased particle volume and geometric diameter of the API particle. In this case, the powder deaggregation mechanism illustrated in Figure 6a upon aerosolization is of great importance to break the powder agglomerates and release the API fines. Studies have indicated that collisions between the agglomerates and the capsule in the flow field are conducive to the breakage of agglomerates [95]. Factors like impact velocity [96] and impact angle [97,98] are other factors that affect agglomerated powder facture tremendously.

**Table 3:** Summary of dry powder-related CMAs and their influence on CQAs and QTPP of cDPIs.

Dry Powder Related CMAs	CQAs	QTPP	Comments	References
API particle morphology	Powder dispersion and de-aggregation	Target drug effects in deep lung	Higher porosity of the API particles gives more lung deposition	[99]
API particle size	Powder dispersion and de-aggregation	Target drug effects in deep lung	Smaller size API particles gives more lung deposition	[90,93]
API particle density	Powder dispersion and de-aggregation	Target drug effects in deep lung	API particles with lower density gives more lung deposition	[91]
API/carrier hygroscopicity	Powder dispersion, de-aggregation, and detachment from carriers	Target drug effects in deep lung	Particle interactions may be dominated by the action of capillary forces in powder systems if humidity has surpassed critical levels	[100]
API particle electrostatic charging	Powder dispersion and de-aggregation	Target drug effects in deep lung	API particles with lower charge gives more lung deposition	[49]
API stability	Effective dose delivery	Ensuring therapeutic effect, avoiding side effects	API particles with better stability gives more effective dose delivery	[101]
API impurity	Effective dose delivery	Ensuring therapeutic effect and avoiding side effects	API particles with less impurity gives more effective dose delivery	[102]
API/carrier surface roughness/rugosity	API dispersion and detachment	Target drug effects in deep lung	API particles with rougher gives more lung deposition; the decrease of the surface roughness of lactose carrier particles in terbutaline sulfate delivery case gives more lung deposition	[103,104,105]
Carrier electrostatic charging	API dispersion and detachment	Target drug effects in deep lung	API particles with lower charge gives more lung deposition	[49]
Carrier particle shape	API dispersion and detachment	Target drug effects in deep lung	The values of either the surface factor or the elongation ratio of lactose in direct proportion to the dispersibility of salbutamol sulfate	[106]
Carrier crystallinity/polymorphs	API dispersion and detachment	Target drug effects in deep lung	α-lactose monohydrate has better performance than anhydrous β-lactose	[107]
Carrier impurity	API dispersion and detachment	Target drug effects in deep lung	Impurities may be responsible for an increase in the adhesive forces between drug and carrier particles	[108]
Carrier particle size	API dispersion and detachment	Target drug effects in deep lung	Reduction of carrier particle size has been proved to ameliorate the aerosolization of various drugs. However, the too-small carrier can also lead to poor flow properties in the powder due to stronger charging interactions caused by increased surface area	[70,109,110]

#### 3.3.2. Carrier-Based Systems

In order to facilitate the API de-agglomeration and release carrier-based formulation systems are used [111]. When it comes to the carrier-based formulation systems, the capsule-derived dispersion of the drug-carrier system is linked with drug-capsule wall collisions, turbulence [112], shear stress during turbulence [113], as well as impactions of particle-particle collisions [13]. Especially the wall collision is an important step for drug detachment from the coarser carrier due to higher kinetic energy during aerosolization [114] (Figure 6b). Usually, the drug-carrier adhesive force contains a combination of forces such as the van der Waals forces (the most substantial forces), electrostatic forces, interlocking forces, and capillary forces [115]. Chemical forces like hydrogen bonding and acid-base interaction forces have also been reported [116]. Considering the effect of carrier particle-wall impaction on dry powder detachment from the carrier, the determination of the detachment efficiency is evaluated by the balance of adhesion energy and impact energy. Therefore, a primary factor related to powder physicochemical property is the adhesion energy of the dry powder, which is inversely proportional to dry powder detachment. Less the contact area between the micronized drug powder and carrier (e.g., increased roughness of dry powder particles, lower specific surface area), the decreased surface energy of the contiguous surfaces, and lower electrostatic charges of dry powder particles, contribute to the reduction of the adhesion force or energy [117].

Furthermore, in addition to dry powder detachment, there is a re-attachment mechanism when the carrier itself collides with the capsule wall that adhered to dry powder fines from a previous collision [118]. Taking all into consideration, physicochemical properties (e.g., polymorphic form, size, etc.) of the carrier excipient are CMA that need to be considered in the same way as the CMA of the API as they are critical for the reproducible performance of a cDPI as defined in the QTPP (Table 3). Though the types of carrier excipients have been thought to affect the charge deposition for dry powder [110], the most commonly used carrier excipient is lactose, thereby only lactose will be discussed in this section. Lactose for inhalation purposes is available in multiple grades, particle size/size distribution, shapes, and surface properties. The selection of the lactose carrier depends on the API particle properties and should be carefully investigated during the formulation development.

In all, the dry powder related CMAs lead to the finding that in the carrier-free formulation, dry powder with required physicochemical properties (e.g., small size, low density, high roughness, low specific surface area, low electrostatic charges, and relatively aspherical shape) are thought to be important determinants for deep lung deposition. With the introduction of carrier-based formulation into dry powder delivery, the selection of the carrier excipients (e.g., lactose) requires careful investigation regarding their physicochemical properties (proper size range, crystalline form, relatively spherical shape, high smoothness) to guarantee QTPP/CQAs achievement.

## 4. Critical Process Parameters (CPPs) in cDPIs

Process understanding through the identification of influential input operating parameters that potentially impact CQAs is a critical part of the pharmaceutical development process. The assessed variables with criticality are CPPs. Based upon the CMAs identified above in cDPIs, CPPs associated with the CQAs of cDPIs are capsule processing and formulation processing.

### 4.1. Capsule Manufacturing

**Figure 7:**
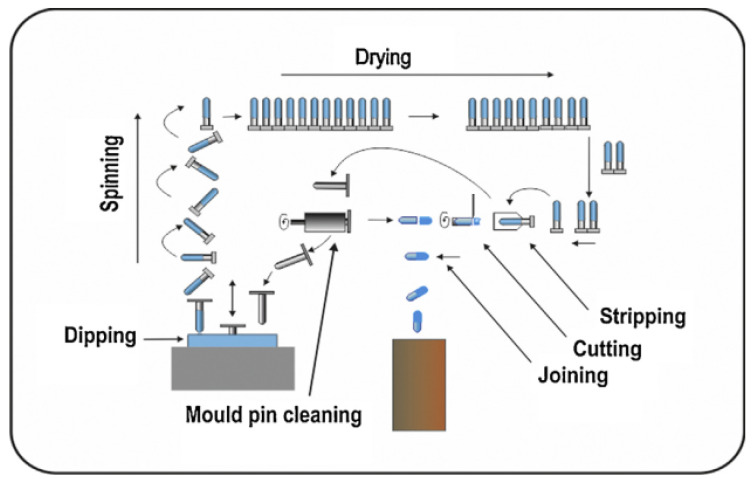
Hard capsule manufacturing. Reproduced with the permission from [120], Elsevier, 2016.

To manufacture gelatin hard capsules for use in DPIs, the first step is to prepare a gelatin solution in demineralized water at a temperature between 60 and 70 °C, which is sufficient to dissolve gelatin and prevent microbial growth [119]. A 30–40% *w*/*w* gelatin solution is viscous and thus is prepared under vacuum to prevent the formation of bubbles. At this step in the process, colorants, and other processing aids (e.g., sodium lauryl sulfate to reduce surface tension) may be added. Viscosity is a critical parameter for determining the capsule wall thickness, which can potentially affect some CQAs by changing the hardness or piercing performance of the capsule. The gelatin solution is fed continuously into the dipping dishes, and standardized steel pins in rows are dipped into the temperature-controlled solution at a predetermined depth. The dipping process is designed so the caps and bodies of the capsules are produced simultaneously. After dipping, the bars are rotated to facilitate even distribution of the gelatin solution around the steel pins, and then the pins undergo several drying stages to achieve the target moisture content [42]. The caps and bodies are then stripped from the pins and trimmed to the appropriate length before being joined together [63]. The process can be visualized in Figure 7.

The manufacturing process of the HPMC capsule containing a gelling system is similar to that of gelatin capsules following the method developed by Eli Lilly, but other methods have been developed [121]. Unlike gelatin solutions, HPMC, gelling system solutions must be of higher temperature (70 °C) to form a film. Due to the poor film formation of HPMC solutions, the success of the manufacturing process of HPMC capsule shells is essential to have a gelling agent (e.g., carrageenan, Pectin, and glycerin, gellan gum) if using the conventional dip molding process similar to hard gelatin capsule shells [122]. Carrageenan is a family of linear sulfated polysaccharides extracted from red edible seaweeds, which can interact with two molecular chains of HPMC in a three-dimensional to form a double helix structure giving a high gel strength to capsules if the auxiliary for gelation contains potassium ion [123]. Specifically, Shionogi Qualicaps Co. (Osaka, Japan) and Zhejiang LinFeng Capsules Co. Ltd. (Shaoxing, China) applied this method and developed their market product Quali-V^®^ HPMC capsules and VegiCaps^®^ Natural Plant Capsule HPMC capsules, respectively. Gellan gum is a water-soluble anionic polysaccharide generated by the bacterium *Sphingomonas elodea*, which can produce the HPMC capsule together with sodium citrate or ethylenediamine tetra acetic acid as a gelling promotor [124]. Vcaps^®^ HPMC capsules from Capsugel (now is Lonza Company, Basel, Switzerland) are manufactured based on this gelling aid. Pectin and glycerin used for gelling with the presence of glacial acetic acid, calcium gluconate, as well as sucrose fatty acid ester are patented by Suheung Capsule Co., Ltd. which later is utilized to produce Embo Caps-Vg^®^ HPMC capsules [125] (Table 4).

A second approach used to manufacture HPMC capsules does not require a gelling system. Rather, the process occurs by dipping hot steel pins into room temperature HPMC solution (i.e., the opposite of cold-gelling, where cold steel pins are dipped into hot HPMC solution) [47]. Different from gelatin, which is liquid at high temperatures and gels when passed through a lower temperature [127], HPMC solutions undergo a sol-gel transformation at a temperature specific to the type of HPMC used [128]. This process of using hot steel pins dipping into HPMC solution at room temperature is known as thermal-gelling [129].

A study including four different capsule formulations’ impact on salbutamol was performed across a stability program [130]. Seven distinct factors were measured which all contribute to the effectiveness of the formulation delivery. The results showed a capsule shell formulation of HPMC that included a gelling agent (carrageenan) and an additional plasticizer (PEG-3350) performed better than other more standard capsule shells with regards to FPF and drug powder retention [130]. Therefore, any variables within HPMC capsules manufacturing must be considered as CMA due to a potential effect on CQAs of the cDPIs.

### 4.2. Capsule Coating Involvement

The final step in manufacturing the hard shell capsules is the removal of the caps and bodies from the steel pins after drying [63,119]. To facilitate this process and ensure the capsule pieces easily slide off, the steel pins are coated on the interior surface with a surface lubricant to function as a release aid [51,120]. The amount of lubricant utilized in the process was not considered to alter the aerosolization properties of powders from cDPIs [80]. The findings from Saim and Horhota determined lubricant levels affect drug retention in the gelatin capsule. The authors hypothesized the effect was related to surface heterogeneity on the internal surface of the gelatin capsule, and there was a range where the lubricant thickness was small enough for particles to become entrapped within the irregular surface [51]. Diez et al., tested salbutamol sulfate (200 mcg), salmeterol xinafoate/fluticasone propionate (100/200 mcg), and budesonide/formoterol fumarate (400/6 mcg) with four internal lubricant variations on the pin bars during HPMC capsule manufacturing [131]. FPD was measured at ambient conditions and after an accelerated stability protocol. Differences were noticed in the aerosolization results which indicate the four internal lubricant levels of the inhalation capsules for certain formulations could be customized to optimize performance for emitted dose, fine particle fraction, and mass media aerodynamic diameter [131]. Saleem et al., suggested that there should be an optimal range of surface lubricant to achieve good powder release from HPMC capsules (measured by drug deposition, ED, and FPF) [80]. The authors also proposed this effect was related to the internal capsule surface homogeneity, where higher lubricant levels led to a smoother internal surface and better aerosolization, since the internal surface lubricant content could potentially be purposely altered to influence capsule-based DPI powder characteristics, as well as abating the electrostatic forces between the capsule and dry powder [132]. Besides the inner lubricant level, the moisture content of the capsule is an important factor responsible for powder retention. It was found that a lower lubricant content reduces powder retention, while low capsule moisture leads to higher powder retention inside the capsule [133].

Differently, Desmond et al., coated the interior surface of the capsule with magnesium stearate (MgSt), which can form stronger Langmuir type films on surfaces potentially sequester the direct contact between formulation powder and capsule [134,135,136,137]. They used two capsule-based DPIs, the Rotahaler^®^, and the Aerolizer^®^, and found that the retained powder in the capsule largely decreased with a concentration of 0.3 g/mL MgSt in Aerolizer^®^ and 0.05 g/mL in Rotahaler^®^ [49]. The enhancement effect on powder emptying was also verified by Srinivas et al., who found that capsules coated with MgSt had a decrease in capsule drug retention compared to uncoated [76]. Interestingly, the same effect was found if using excipient enhanced growth (EEG) [138,139] particles or L-leucine, indicating that any substance with a lubricant nature may reduce capsule retention [76]. Lastly, it was thought theoretically that the particle size distribution of the lubricant used in carrier-based formulations would affect detachment and de-agglomeration and cause fluctuations in capsule retention, however, this did not show in the experiment [76].

In addition to interior coating, capsule exterior coating is another distinctive capsule coating strategy, which reduces the electrostatic forces between capsule and DPI. One representative study by Srinivas et al., exhibited that coating the exterior surface of the capsule and the interior surface of the inhaler with the same commercial polytetrafluoroethylene (PTFE) suspension (LU™708, Sprayon Products, Cleveland, OH, USA) can dramatically decrease the drug retention of the capsule, inhaler as well as the mouthpiece. While the detailed mechanism remains unknown [76], PTFE is a special material with low surface energy (high contact angle) with notorious non-stick properties as well. Whether or not other coating materials can also function in the same manner is yet to be explored.

### 4.3. Capsule Filling Control

#### 4.3.1. Filling Method

Dry powder must be metered into a capsule to allow application of the product by an inhalation device. On a laboratory scale, a capsule is usually filled manually. For clinical and commercial purposes, specialized dosing equipment is required for capsule filling due to the low fill weights in each dose. Dosator technology is the most traditional automated filling system, which consists of mechanical compaction-based dosator systems and vacuum compaction-based dosator systems [10,140]. The compaction of powder leads to the increase of bulk density of the powder, which highly affects powder flowability [141], serving as the disadvantage of traditional dosator systems. Recently microdosing dosator filling has been developed which does not require compaction. A study by Faulhammer et al., investigated the CPP of the microdosing comparing the performance of different types of powders. The study provided evidence that the accurate filling of 1 -45 mg of DPI powders into capsules depends on processing parameters that can be adjusted for the different types of powders [142]. Different from dosator systems, tamp filling system is an automated filling system requiring a lower degree of formulation compaction, and is very suitable for loading highly potent compounds [143]. A more recent capsule dosing technology is based on ‘pepper-shaker’ or ‘pepper-pot’ principle [144]. This method fills the capsule by tapping the powder in a pepper-shaped shaker or pot, without the consolidation of powder to transfer the powder to the capsule. Importantly, it can precisely fill the capsule with a dose as low as 10 µg, which is perfect for the expensive or potent drug [10]. An additional benefit can be its ability to be fitted with a relative humidity control unit, thus decreasing aggregates formation when the ambient humidity is too high [10]. However, the limitation of this technology is the limited throughput which is limited to 600 capsules/h [145].

#### 4.3.2. Filling Weight Variability

For automated machine filling, powder flowability is a critical factor to achieve consistent fill weight [146]. Faulhammer et al. conducted a comprehensive DoEs study to understand process parameters involved in dosator capsule filling process that influence filling weight variability [147]. In their study, different conclusions can be obtained on the correlation between filling weight variability and capsule filling method parameters with the use of powder of different properties. Large particles with high density exhibited an inverse correlation between filling weight variability and capsule filling method parameters (the dosator diameter, filling speed, and the powder layer depth). While for low dense particles with a particle size less than 10 µm, high filling speed resulted in high filling weight variability. In an additional study, they found a correlation between the capsule filling weight and the particle size, the air permeability, and the compressibility. For lower dosed powders, additional critical factors were observed like the wall friction angle, the tapped density, and the particle shape proved to be important factors [142]. These studies were complemented by Stranzinger et al., who investigated that higher filling speed, the compression ratio of dosing chamber to powder bed height (1:1, rather than > 1:1), and smaller dosator size can result in an increasing filling weight variability in the case of low bulk density (<45 g/mL) lactose with the particle size <10 µm [148]. Furthermore, Llusa et al., proved that high filling weight variability can result from the vibrations of the capsule-filling machine [149]. Therefore, minimizing the vibrations of the capsule-filling machine can improve filling weight variability.

#### 4.3.3. Capsule Fill Weight Effects

The mass of dry powder filled into capsules is typically dependent upon the desired dose to be administered to patients; however, this may also have effects on the aerosol performance of the formulation. The weight of drug/formulation powder filled into the capsule causes the increase of the capsule weight initially, which contributes to the gravitational force of the filled capsule. Therefore, filling amount variation is thought to affect capsule motion profile within cDPIs, since capsule motion profile should be the outcome of the interplay between the aerosol flow force and the gravitational force of the filled capsule [79]. Heavier capsules tend to fall instead of moving inside the inhaler during aerosolization in comparison to lighter capsules. This tends to lower the velocity of the capsule movement [79]. As the powder is emitted from the capsule during inhalation, the capsule weight decreases second by second. This dynamic transformation leads to the continuous alteration of the gravitational force of the capsule. It is difficult to say at what point in time the scale changes enough to be negated by the forces due to airflow. When the initial powder filling weight is low, the changes in forces due to gravity can be negligible relative to airflow forces throughout an inhalation compared to higher fill weight capsules [147]. The degree to which there is a transitional difference remains to be investigated. One study from Ashkan et al., studied the effect of capsule filling weight on in vitro aerodynamic performance. They filled the capsules with different weights of micronized ibuprofen (10 mg, 25 mg, and 50 mg). As a result, they found that the ED value was independent of the alteration of capsule filling weight, while the increase in capsule filling weight led to largely decrease of FPF values [150].

### 4.4. Capsule Storage Control

It is well known that ambient factors can cause the denaturation of gelatin capsules. This is mostly due to the nature of gelatin, which can have decreases rigidity from undergoing gamma radiation [151], increases brittleness from incompatible solvents [152,153], hygroscopic fill [154], and is incompatible with reducing sugars, plasticizers, and preservatives [155]. In addition, having the proper relative humidity (RH) for storage is of great significance. RH can potentially change the original stiffness or hardness of the capsule [156]. Too low in RH gives rise to capsule brittleness [156], but too high of an RH brings about capsule stickiness [46,157]. Several studies have suggested that there was a correlation between the ambient moisture content and the piercing profile of the capsule in the device. Most notably, a higher penetration force was needed to puncture capsules stored in lower moisture environments for both HPMC and gelatin capsules [65]. Furthermore, Coulman’s study showed a higher percentage of gelatin capsules with a ‘regular’ shaped puncture after removing the angular pin from punctured capsules if stored in desiccators with Mg (NO_3_)_2_ to create higher moisture contents. But no significant differences have been found between different HPMC capsules [58]. Also, RH within different capsules can play a role in the fluidization and dispersion of powders from within capsules. The formation of liquid bridges between formulation particles as well as the capsule wall results from high RH. These liquid bridges create a binding capillary force that can lead to increased powder retention [158,159,160].

In addition to RH, the ambient temperature also has the potential to fluctuate capsule stiffness and hardness [156]. Within Coulman’s study, the author also simply looked at the relationship between the storage temperature and the puncturing characteristics, indicating that higher storage temperature tended to result in lower puncture forces as well as larger capsule puncture areas [58]. These reasons further add to the necessity for maintaining the storage conditions for capsules at reasonable moisture and temperature levels regarding strong quality control.

At the aspect of some preloaded capsules, chemical interaction may occur during storage if without consideration of incompatibility between capsule shells material and formulation components. An example is a highly moisture-sensitive drug, salicylic acid, which will degrade at high ambient humidity. One study found that the amount of salicylic acid degradation of salicylic acid was higher when stored in gelatin capsules vs. HPMC capsules at the initial time point (only slightly higher in gelatin than HPMC) and at 3, 6, 12, and 18 months when stored at 25 °C at 60% relative humidity [161]. One explanation for this occurrence is the significantly higher moisture content in gelatin capsules versus HPMC hard capsules. Therefore, in cases where the drug is extremely sensitive to moisture, HPMC-based capsules may provide a better option for storage [62]. Also, when loading drugs that have a high sensitivity to oxidation careful attention needs to be made for capsule selection as HPMC capsules are known to be more permeable to oxygen than gelatin capsules. When comparing two films of HPMC and gelatin, each 100 µm in thickness, the observed oxygen permeation values were 166 cm^3^/m^2^/day and 3.41 cm^3^/m^2^/day, respectively. This demonstrates a substantial difference that could ultimately affect the potency of the drug [62]. While this oxygen permeation can be a potential pitfall for the drug product, this effect can be mitigated by utilizing proper packaging and storage of the capsules before delivering the dose (e.g., the capsules can be stored in blister packaging and opened before administration).

This suggests that CPPs for inhalation capsule manufacturing involve several potential factors to be investigated like capsule coating, capsule filling method, capsule filling weight, weight variability, and storage RH and temperature control should be carefully considered in cDPIs product development.

## 5. Conclusions

The QbD framework is a valuable tool in pharmaceutical development for several years, which has been applied in many dosage form development programs. For the DPI product development, the scientific literature on QbD is still sparse. The major challenge for such products remains the multifactorial nature and complex interaction between formulation, primary packaging, and the device component. This review intends to provide information about aspects regarding the capsule component to be assessed when performing a QbD approach for cDPI products (Figure 1). In this work, we explored the interaction between capsule material properties, powder formulation properties, and device engineering and design which might have a direct impact on the product performance and hence, the therapeutic effect. Identification and understanding of the CQAs and CMAs will enable successful development and manufacturing in accordance with the QTPP. It will contribute to streamlining and de-risking new product development efforts in this field. Finally, it might serve as a roadmap for new and generic developments and support regulatory decisions, given the complexity of cDPI systems.

## Figures and Tables

**Table 1:** Examples of capsule-based DPIs available on the U.S. market and the drug(s) delivered by the device. Included also are the U.S. marketed products that utilize each device (if any) and the capsule in the FDA-approved formulation.

Capsule-Based DPIs	Drug(s) Delivered	U.S. Marketed Product	Capsule Types	References
Aerohaler^®^	Ipratropium bromide	-	-	[8,11,12]
Aerohaler^®^/Cyclohaler^®^	Formoterol fumarate *	Foradil^®^ Aerohaler^®^	Gelatin	[8,11,12,13,14,15]
	Salbutamol sulfate			
	Beclomethasone			
	Dipropionate			
	Ipratropium bromide			
	Budesonide			
	Formoterol			
Eclipse^®^	Sodium cromoglycate	-	-	[13,15]
FlowCaps^®^	N/A	-	HPMC	[13]
HandiHaler^®^	Tiotropium bromide	SPIRIVA^®^ HandiHaler^®^	Gelatin	[8,11,12,13,15]
Inhalator^™^	Fenoterol	-	-	[11,12,13]
Podhaler^™^	Tobramycin	TOBI^™^ Podhaler^™^	HPMC	[8,15]
Rotahaler^®^/DPIhaler^®^	Salbutamol sulfate	-	-	[8,11,12,13]
	Beclomethasone			
	Dipropionate			
RS01	Mannitol	Aridol^®^	Gelatin	[16,17]
Spinhaler^®^	Sodium cromoglycate	Intal^®^ Spincaps^® †^	Gelatin	[8,11,12,13,15,18,19]
Turbospin^®^	Colistimethate sodium	-	-	[8,15]
Neohaler^®^	Glycopyrrolate	Seebri^™^ Neohaler^®^	HPMC	[8,15]
	Indacaterol	Arcapta^®^ Neohaler^®^	Gelatin	

* Indicates the drug present in the U.S. marketed product. ^†^ no longer available on the U.S. market.

**Table 2:** Side-by-side comparison between the different capsule materials.

Properties	Capsule Material	
	Gelatin	HPMC
Moisture content	13–16%	Less than 9%
Moisture transfer	Hysteretic	Sensitive
Required puncture force	More	Less
Aperture shape after piercing	Irregular	Regular
Shedding of pieces after piercing	More	Less
Brittleness	More brittle	Less brittle
Generation of “flap” after piercing	More	Less
Capsule filling	Easy	Relatively hard
Powder retention after inhalation	High	Low
FPF after inhalation	High	Low

**Table 4:** Information on the empty HPMC capsules and their manufacturer, reprinted from [126], Canadian Society for Pharmaceutical Sciences 2010.

Capsule Shell Brand	Name Manufacturer	Registered Year in the U.S.	Gelling Aid
Quali-V	Shionogi Qualicaps	July 2002	Carrageenan
Vcaps Plus	Capsugel (A division of Pfizer)	-	None
Vcaps	Capsugel (A division of Pfizer)	April 2003	Gellan gum
VegiCaps	G S Technologies Inc. (now R.P. Scherer Technologies ownership)	May 1989	None
Embo Caps -Vg	Suheung Capsule Co., Ltd.	-	Pectin and glycerin
Capstech’s HPMC Capsule	Baotou Capstech Co., Ltd.	-	None
Natural Plant Capsule	Zhejiang LinFeng Capsules Co. Ltd.	-	Carrageenan
